# Epidemiology of malaria in an area prepared for clinical trials in Korogwe, north-eastern Tanzania

**DOI:** 10.1186/1475-2875-8-165

**Published:** 2009-07-18

**Authors:** Bruno P Mmbando, Method D Segeja, Hamisi A Msangeni, Samwel H Sembuche, Deus S Ishengoma, Misago D Seth, Filbert Francis, Acleus S Rutta, Mathias L Kamugisha, Martha M Lemnge

**Affiliations:** 1 National Institute for Medical Research, Tanga Medical Research Centre, PO Box 5004, Tanga, Tanzania; 2 Department of Biostatistics, University of Copenhagen, DK-1014 Copenhaven K, Denmark

## Abstract

**Background:**

Site preparation is a pre-requesite in conducting malaria vaccines trials. This study was conducted in 12 villages to determine malariometric indices and associated risk factors, during long and short rainy seasons, in an area with varying malaria transmission intensities in Korogwe district, Tanzania. Four villages had passive case detection (PCD) of fever system using village health workers.

**Methods:**

Four malariometric cross-sectional surveys were conducted between November 2005 and May 2007 among individuals aged 0–19 years, living in lowland urban, lowland rural and highland strata. A total of 10,766 blood samples were collected for malaria parasite diagnosis and anaemia estimation. Blood smears were stained with Giemsa while haemoglobin level was measured by HaemoCue. Socio-economic data were collected between Jan-Apr 2006.

**Results:**

Adjusting for the effect of age, the risk of *Plasmodium falciparum *parasitaemia was significantly lower in both lowland urban, (OR = 0.26; 95%CI: 0.23–0.29, p < 0.001) and highlands, (OR = 0.21; 95%CI: 0.17–0.25, p < 0.001) compared to lowland rural. Individuals aged 6–9 years in the lowland rural and 4–19 years in both lowland urban and highlands had the highest parasite prevalence, whilst children below five years in all strata had the highest parasite density. Prevalence of splenomegaly and gametocyte were also lower in both lowland urban and highlands than in lowland rural. Anaemia (Hb <11 g/dl) prevalence was lowest in the lowland urban. Availability of PCD and higher socio-economic status (SES) were associated with reduced malaria and anaemia prevalence.

**Conclusion:**

Higher SES and use of bed nets in the lowland urban could be the important factors for low malaria infections in this stratum. Results obtained here were used together with those from PCD and DSS in selecting a village for Phase 1b MSP3 vaccine trial, which was conducted in the study area in year 2008.

## Background

Malaria remains a major cause of morbidity and mortality in sub-Saharan Africa [1]. Worldwide malaria morbidity is estimated to be 300–500 million cases and about 1 million deaths each year, of which 90% occur in sub-Saharan Africa [2,3]. The vast majority of deaths occur among children below five years of age and pregnant women, especially in remote rural areas with poor access to health services [4].

In many parts of the world, adequate treatment of malaria is becoming increasingly difficult due to worsening problems of drug resistance, which have rendered cheap drugs such as chloroquine and sulphadoxine/pyrimethamine ineffective [5,6]. Hence, prevention of malaria by vaccines is perceived as a tool that will compliment currently available strategies for malaria control [7]. With more malaria vaccine candidates becoming available [7,8], a suitable chosen area with detailed epidemiological information is required for testing and evaluating different trial endpoints [1,9]. The site needs to be prepared and should focus on characterising the population (*eg *size, structure), accessibility of the site, availability of health services and malaria burden in the area. Site preparation involving local communities is also likely to reduce ethical issues which could otherwise compromize implementation of the trials [10].

In 2004, the African Malaria Network Trust (AMANET) received a grant from AIDCO, which, among other things, was to support capacity strengthening of selected African Research institutions to be able to conduct malaria vaccine trials. Site characterization for future trial sites was one of the most important components of the grant. The National Institute for Medical Research at its Centre based in Tanga, Tanzania, was one of the beneficiaries of the sub-grants given by AMANET. One of the objectives of the project was thus to conduct repeated malariometric surveys in 12 villages to describe distribution of malaria parasites as well as anemia and splenomegaly. This was done during short and long rainy seasons in urban and rural areas of Korogwe district between November 2005 and May 2007. Results from this study were used together with other data to provide guidance in the selection of villages suitable for conducting malaria vaccines

## Methods

### Study area and population

The study was conducted in 12 villages of Korogwe district, Tanga region, north-eastern Tanzania. The population of Korogwe district was estimated to be 261,004 in 2002, with a growth rate of 1.4% per annum [11]. The district has 47 dispensaries, four health centres and two hospitals. Korogwe district can be topographically stratified into lowland and highlands zones, with altitudes ranging from 300–1,200 meters above sea level (mASL). Malaria transmission in these areas decreases with increasing altitude and varies within a short distance [12-14]. In both zones, malaria transmission is highest during and following the long rainy season, which usually extends from March through July [15]. Low transmission is experienced during short rains (October – December).*Anopheles gambiae *s.s and *Anopheles funestus *are the main vectors of malaria [16]. *Plasmodium falciparum *is the predominant malaria species accounting for more than 90% of all infections, the rest being *Plasmodium malariae *and *Plasmodium ovale *[17].

**Figure 1:**
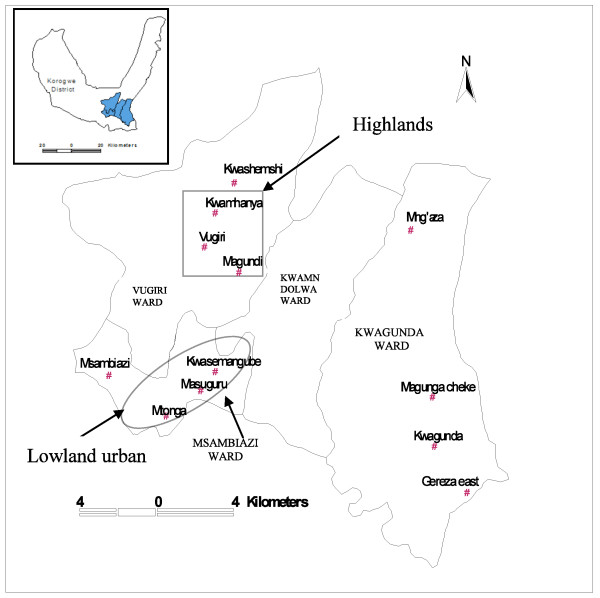
**Map showing study area**. A box defines villages in the highlands stratum; oval is the villages in urban and the rest are villages in the lowland rural stratum.

The 12 study villages are grouped into three strata namely: lowland rural, lowland urban and highlands (Figure 1). Altitude of the lowland stratum ranges from 300–410 mASL and that of highlands spans between 600–1,000 mASL. The lowland urban stratum was defined as villages or communities living within administrative areas designated as urban according the Tanzanian census of 2002 [11]. Two villages in lowland rural (Kwashemshi and Mng'aza) and two in highlands (Magundi and Kwamhanya) have been implementing passive case detection (PCD) of febrile episodes using community resource persons (CORPs) since January 2006. In the PCD system, CORPs manages uncomplicated malaria cases at the village level using first line anti-malarial drug.

### Study design

The study involved four cross sectional malariometric surveys which were conducted during short rains in November/December 2005 and 2006, and long rains in May/June 2006 and 2007. The study involved individuals aged 0–19 years who were randomly selected from the census database during the first baseline survey. Prior to the study, a population census was conducted in October/November 2005 followed by demographic surveillance system (DSS) follow ups which are being implemented since January 2006. Population of individuals below 20 years was estimated to be 5400, lowlands rural, 6200 lowland urban and 2100 in the highlands. A socio-economic survey was conducted as part of the DSS between January and May 2006. The sample size was calculated based on population survey design in each stratum. At 95% confidence interval and 80% power, a sample size required was 985, 1130 and 385 in rural lowland, lowland urban and highlands, respectively. A list of individuals to participate in the surveys was randomly generated from the census database.

### Ethical consideration

Ethical clearance was granted by the Medical Research Co-ordinating Committee of the National Institute for Medical Research, Tanzania. Prior to the study, sensitization meetings were held in each village to explain the objectives and methodology of the study as well as seeking for community consent. Informed consent was obtained both orally and in writing from all individual participants or parents/guardians in case of children during the survey.

### Clinical examination, blood collection and laboratory analysis

Demographic information of each selected individual including name, sex, age and village of residence were recorded on a morbidity questionnaire. Thereafter, clinical and physical examination was done to assess participant's general clinical condition. Assessment of spleen enlargement was done according to Hackett's classification. Axillary temperature was measured using electronic thermometer. Individuals found to be sick were treated appropriately or referred to the nearby health facilities depending on the patients' clinical condition.

A blood sample was collected for thick and thin smears preparation for malaria parasite quantification and identification, haemoglobin estimation and for other bioassays. Haemoglobin levels were estimated using HemoCue machine (HemoCue, Ångelholm, Sweden). Blood smears were stained with Giemsa and asexual parasites were counted against 200 white blood cells (WBCs) while sexual parasites were counted against 500 WBCs. A blood slide was declared negative after reading 100 high power fields. For quality control, 10% of the blood smears were randomly selected and read by another microscopist. Smears with discordant results were re-read by a third microscopist. Results of two microscopists with difference of less than 20% were adopted according to the standard operating procedures.

### Data management and analysis

Data were double entered and verified in Microsoft Access, while consistence checks and analyses were done using STATA software version 8.0 (StataCorp LP, TX, USA). Principal component analysis (PCA) was used to categorize households into different socio-economic scores. Household scores were grouped into three categories at ratios 40:40:20 as low, medium and high SES's [18]. Variable which were considered in the PCA were: type and material used to build the house, source of power for lighting and cooking, ownership of radio, bicycle and mobile phone, ratio of number of sleeping bedrooms and bed nets to number of household dwellers, occupation of head of household as well as number of animal and size of land owned or cultivated by the family. About 50% of individuals had two or more observations in the four surveys and, therefore, generalized estimating equation (GEE) with robust standard errors was used to assess risk factors associated with the response variables. Response variables modeled using GEE were prevalence of parasitaemia, anaemia (<11 g/dl), splenomegaly, gametocytes and mean haemoglobin concentration (g/dl). Haemoglobin concentration was adjusted for the physiological effect of oxygen-carrying capacity as a result of the increase in altitude [19]. Parasite counts/200WBC were converted into parasites/μL by multiplying by 40, assuming that 1 μL of blood (of a physiologically normal person) contains 8,000 WBCs, and the counts were transformed to normal using natural logarithm. Anaemia was defined as Hb <11 g/dl whilst splenomegaly was referred to as presence of any enlarged spleen detectable during physical examination. Data from similar season (short or long rains) were pooled during analysis. P-value below 0.05 was considered significant.

## Results

### Socio-demographic characteristics

Table 1 shows socio-demographic characteristics, altitude and pooled malaria prevalence of the study villages. A total of 10,851 observations were done among 6,375 individuals who were seen during the four rounds surveys. The altitude of the village in lowland urban was similar to that of lowland rural. Households in lowland urban had higher socio-economic scores while those in the highlands had the lowest. Bed net coverage was also higher in the lowland urban, a similar trend to that of socio-economic scores. Overall parasite prevalence was highest in lowland rural, and had an inverse relationship to that of bed net coverage and that of SES.

**Table 1:** Characteristics of study villages and malariometric indices of individuals sampled into surveys done during short and long rain seasons, Nov/Dec 2005 – May/Jun 2007.

Strata, village name (observations)	Altitude, mean (range), m	Median SES score (IQR)	Median age (IQR), years	Bed net use, %	Parasite prevalence, %
**Lowlands rural (4,434)**					
Magunga cheke (566)	328 (286–377)	0.28 (-0.34, 0.78)	7.4(3.8, 11.5)	61.7	31.8
Gereza east(455)	316 (278–333)	-0.11 (-0.53, 0.33)	8.2(4.8, 12.8)	47.0	57.1
Kwagunda(1,057)	306 (270–328)	0.1 6(-0.45, 0.59)	6.8(3.9, 10.9)	40.7	33.5
Mng'aza^§^(444)	334 (295–400)	-0.51 (-1.08, -0.11)	6.7(3.0, 11.8)	37.1	57.2
Msambiazi(875)	349 (305–382)	-0.56 (-1.38, 0.30)	8.0(3.5, 13.3)	59.5	23.9
Kwashemshi^§^(1,437)	409 (354–580)	-0.37 (-1.01, 0.17)	6.4(3.1, 10.8)	84.6	26.6
**Lowlands urban (4,446)**					
Masuguru (714)	322 (275–345)	1.38 (0.73, 1.96)	7.5(3.7, 12.0)	92.5	4.9
Mtonga(2051)	341 (273–416)	0.58 (-0.14, 1.26)	7.4(3.9, 11.8)	66.9	11.8
Kwasemangube(1681)	356 (310–494)	-0.04 (-0.60, 0.61)	7.2(3.8, 11.4)	49.3	13.4
**Highlands (1,571)**					
Magundi^§^(491)	638 (486–799)	-0.78 (-1.29, -0.27)	9.7(4.5, 13.4)	18.7	12.7
Kwamhanya^§^(345)	761 (590–818)	-0.91 (-1.44, -0.15)	7.9(3.0, 13.3)	42.9	8.7
Vugiri(735)	941(868–1043)	-0.59 (-1.03, -0.03)	7.4(3.7, 11.9)	49.3	12.0

^§^Villages where febrile passive case detection using CORPs strategy was introduced in January 2006.

### Malaria parasite prevalence and density

**Figure 2:**
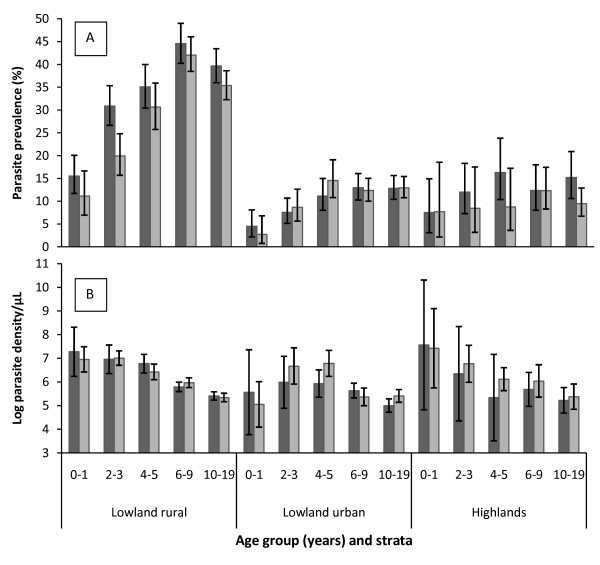
**Prevalence (panel A) and log mean density (panel B) of *P. falciparum *parasites by age-group, strata and season**. Dark grey bars represent short rainy seasons; grey bars are for long rainy seasons, while line segments represent 95%CI.

In the four cross sectional surveys, a total of 10,766 blood samples were collected, 4,778 (44.4%) from lowland rural, 4,424 (41.1%) from lowland urban and 1,564 (14.5%) from highlands. Parasite prevalence during long rain seasons was 32.8% (95%CI: 31.0–34.7) in lowland rural, 11.8% (95%CI: 10.5–13.2) in lowland urban and 10.0% (95%CI: 8.0–12.3) in highlands; while in the short rains it was 35.1% (95% CI: 33.2–37.1) in lowland rural, 10.8% (95% CI: 9.6–12.2) in lowland urban and 13.1% (95% CI: 10.8–15.7) in highlands. The age specific prevalence is shown in Figure 2A. In the lowland rural, prevalence peaked at age group 6–9 years while in lowland urban and highlands it was highest in individuals aged 4–19 years.

**Figure 3:**
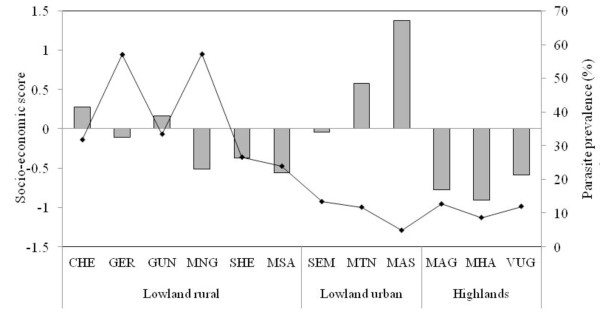
**Distribution of socio-economic scores (bars) and *Plasmodium falciparum *prevalence (line) by village and strata**. Socio-economic data were collected between Jan-May 2006, while malaria prevalence is for Nov/Dec 2005 to May/Jun 2007. Villages which are abbreviated in the x-axis are as follows: Magunga cheke (CHE), Gereza east (GER), Kwagunda(GUN), Mng'aza (MNG), Msambiazi (MSA), Kwashemshi (SHE), Kwasemangube (SEM), Mtonga(MTN), Masuguru (MAS), Magundi (MAG), Kwamhanya (MHA) and Vugiri (VUG).

Adjusting for the effect of age, the risk of *Plasmodium falciparum *parasitaemia was significantly lower in both lowland urban, (OR = 0.26; 95%CI: 0.23–0.29, p < 0.001) and highlands, (OR = 0.21; 95%CI: 0.17–0.25, p < 0.001) compared to lowland rural. Use of bed nets was associated with low parasite prevalence, (OR = 0.68; 95%CI: 0.61 – 75, p < 0.001), whereas individuals from low and medium SES were at higher risk of malaria infection, OR = 2.02 (95%CI: 1.69–2.41, p < 0.001) and OR = 2.47 (95%CI: 2.05–2.99, p < 0.001), respectively, when compared to high SES. Table 2 shows risk factors associated with malaria parasitaemia for each stratum when adjusted for age; where villages with PCD in highlands had significantly lower malaria parasite prevalence, (OR = 0.43; 95% CI: 0.29–0.62, p = 0.001) and that the effect of SES on prevalence was higher in lowland urban and lower in the highlands (see also Figure 3). The effect of season (short rains) was only significantly higher in the lowland rural villages. In all strata, children below five years of age had the highest parasite density (Figure 2B). Mean log parasite density was significantly lower in lowland urban by 0.371 (95%CI: 0.188–0.554, p < 0.001) and marginally significant in highlands (coef = -0.268; 95%CI: -0.539 – -0.002, p = 0.052), compared to lowland rural. *Plasmodium falciparum *was the predominant species accounting for 97.6% of all malaria infections while *Plasmodium ovale *and *Plasmodium malariae *were 0.3% each, and the rest were mixed infections.

**Table 2:** Odds ratios for risk factors associated with presence of *P. falciparum *adjusted for effect of age in each stratum.

Strata	Variable	OR	95% CI		P-value
Lowland rural	SES-medium	1.744	1.371	2.217	< 0.001
	SES-low	1.895	1.484	2.42	< 0.001
	Short rains	1.309	1.168	1.466	< 0.001
	bed net use	0.619	0.543	0.705	< 0.001
	CORPs	1.079	0.928	1.256	0.322

Lowland urban					
	SES-medium	2.299	1.713	3.084	< 0.001
	SES-low	3.896	2.818	5.387	< 0.001
	Short rains	0.970	0.810	1.162	0.740
	bed net use	0.758	0.611	0.939	0.011

Highlands					
	SES-medium	2.118	0.817	5.487	0.122
	SES-low	2.441	0.976	6.109	0.057
	Short rains	1.184	0.877	1.597	0.271
	bed net use	0.694	0.481	1.001	0.051
	CORPs	0.425	0.290	0.623	< 0.001

Overall gametocyte prevalence was highest (4.2%) in lowland rural. In lowland urban and highlands it was 1.2% and 1.3%, respectively. Regression analysis showed lower risk of gametocytaemia in both lowland urban, (OR = 0.367; 95%CI: 0.266–0.505, p < 0.001) and highlands, (OR = 0.383; 95%CI: 0.245–0.600, p < 0.001) compared to lowland rural. The odd ratio of gametocytaemia in individuals from medium and low compared to high SES households were 2.58 (95%CI: 1.59–4.19) and 2.62 (95%CI: 1.58–4.35), respectively. In villages with PCD, the carriage rate was significantly low, (OR = 0.675; 95%CI: 0.472–0.965, P = 0.031). There was no seasonal variation in gametocyte rates.

### Splenomegaly prevalence

Prevalence of splenomegaly during the long rains was 16.5%, 5.3% and 3.5% while in short rain season was 19.1%, 3.9% and 5.0% in lowland rural, lowland urban and highlands, respectively; and there was no difference between the two seasons. Similarly, lower risk was observed in individuals who were using bed nets, OR = 0.62 (95%CI: 0.54–0.71, p < 0.001) and in villages with PCD, OR = 0.42 (95%CI: 0.34–.51, p < 0.001) while higher risk was seen in low SES, OR = 1.82 (95%CI: 1.43–2.32, p < 0.001) and medium SES, OR = 2.29 (95%CI: 1.78– 2.94, p < 0.001).

### Haemoglobin levels and anaemia prevalence

Mean Hb concentration adjusted for effect of age and gender during the long rainy season in three strata was 11.32 g/dL (95%CI 11.26–11.38), 11.51 g/dL (95%CI: 11.45–11.57) and 11.65 g/dL (95%CI: 11.55–11.75), while during the short rains was 11.10 g/dL (95%CI: 11.04–11.16), 11.44 g/dL (95%CI: 11.38–11.49) and 11.07 g/dL (95%CI: 10.96–11.18), in the lowland rural, lowland urban and highlands, respectively. Table 3 shows the results from regression analysis for continuous and binary models for variables associated with variation in mean Hb and prevalence of anaemia. Mean Hb was significantly higher among individuals living in villages with PCD and those using bed nets, and it was lower in those with low SES. Lowland urban had significantly higher mean Hb, (Table 3).

**Table 3:** Coefficients and odds ratios for risk factors associated with variation in mean Hb concentration (g/dL) and anaemia (Hb <11 g/dL) status in the study villages during four malariometric surveys.

	Hb concentration, g/dl				Anaemia, Hb <11 g/dl			
	
Variable	Coef.	95% CI.		P-value	OR	95% CI.		P-value
*P. falciparum*	-0.410	-0.474	-0.347	< 0.001	1.536	1.382	1.707	< 0.001
Stratum-lowland urban	0.153	0.082	0.225	< 0.001	0.740	0.659	0.831	< 0.001
Stratum-highlands	-0.010	-0.101	0.082	0.835	1.106	0.955	1.281	0.177
SES-medium	-0.043	-0.123	0.036	0.285	1.064	0.930	1.216	0.366
SES-low	-0.158	-0.247	-0.069	< 0.001	1.270	1.098	1.470	0.001
Short rains	0.024	-0.018	0.066	0.267	0.984	0.911	1.062	0.676
CORPs	0.294	0.219	0.369	< 0.001	0.642	0.567	0.727	< 0.001
bed net use	0.088	0.037	0.140	0.001	0.955	0.873	1.046	0.322

The estimates are adjusted for the effect of age and gender.

A similar pattern as that of mean Hb was seen for variables associated with risk of anaemia (Table 3). Variables that were associated with increase in mean Hb were also associated with decrease in anaemia prevalence and vice versa, except the use of bed nets which had no effect on the prevalence of anaemia.

## Discussion

This epidemiological study was designed to obtain data on malariometric indices in villages with different malaria transmission intensities during short and long rainy seasons in Korogwe district, north-eastern Tanzania. It was aimed at providing baseline epidemiological information needed in selection of areas for implementing malaria clinical trials and involved both rural and urban settings.

Parasite prevalence is considered as the easiest way of measuring malaria transmission compared to entomological and serology measures. However, it is more affected by seasonal variation in transmission levels, survey timing, and peak transmission seasons, and hence can be seen as reliable within a short time interval [20]. Unlike serology measures which can capture long term transmission [20] and entomological measures which at some points is not possible because of low transmission [15], parasitological measure can be used to assess change in transmission. Data presented here shows that malaria prevalence was lower in the highlands and lowland urban strata, and that the malaria transmission levels were similar in the two strata. Taken together, prevalence data on all malariometric indices and mild anaemia (Hb <11 g/dl), seen in this study, provided a good estimate of malaria endemicity in the three strata.

Previous studies in Korogwe have reported a decrease in malaria with increase in altitude [12,13,17]. However, this study is the first to provide data on malaria epidemiology in a lowland urban area of Korogwe, which found low malaria parasite prevalence and splenomegaly in this stratum when compared to the lowland rural. The low malaria transmisison in lowland urban stratum as shown by low parasite prevalence, splenomegaly, gametocyte carriage, and anaemia could be due to better social economic status, higher bed net coverage and access to better health care. Better houses and access to bed nets provide urban population with reliable means of protection from mosquito bites which cannot be easily accessed by poor communities from rural villages [21,22]. This indicates that the urban population might be exposed to low density of infected mosquitoes per person and therefore these villages have low malaria transmission intensity despite being located in the lowland [23-25].

In the present study, the finding of peak malaria parasite prevalence in children aged 6–9 years in lowland rural, while in lowland urban and highlands it was high in all individuals aged 4–19 years, suggests better immunity in the lowland rural and thus higher exposure to mosquito bites and malaria infection. Notwithstanding this, children below 5 years had the highest parasite density in all strata indicating immature immunity and higher vulnerability to malaria. As expected, children in the low malaria risk area of lowland urban had lowest parasite density. The observation of lack of seasonal variation in prevalence of gametocytes and splenomegaly seems to suggest similar transmission dynamics during short and long rains in the 2 year study period. Furthermore, the significantly higher malaria parasite prevalence observed in lowland rural during the short rains could be due to increased rainfall in October/November 2006.

Data from this study found significant variation in malaria transmission within a short distance; and this might have a bearing on malaria diagnosis and management. Findings from this study suggest that trials aimed at higher malaria transmission areas should focus in the rural areas where socio-economic status is low. And socio-economic status remains an important risk factor for malaria transmission, and so efforts to combat malaria should also focus on socio-economic status of the communities.

## Conclusion

This study has documented low level of malaria infections (prevalence, gametocyte carriage, splenomegaly and anaemia) in the lowland urban villages, which could be due to different factors; some of which could not be fitted or explained in this paper and therefore needs more exploration. Use of sensitive PCR methods for detection of asexual and sexual parasites to rule out sub-microscopic parasitaemia is recommended. Effect of PCD in the area was found to have led to a significant reduction in prevalence of both asexual and sexual forms of *P. falciparum *as well as increasing mean Hb concentration. Data from this study and others on the immunology of malaria (Segeja *et al *submitted for publication), DSS, and PCD were used in the selection of a study site for a Phase Ib malaria vaccine trial of MSP3 (Lusingu *et al*, in press). The data will also be useful in planning and implementing other trials in Korogwe district, such as a Phase 3 of RTS,S vaccine trial.

## Abbreviations

PCD: passive case detection; CORPs: community resource person(s); mASL: meters above sea level; % percent; < less than; dL: decilitre; OR: odds ratio; AMANET: African Malaria Network Trust; MSP3: merozoite surface protein 3; DSS: demographic surveillance system; μL: microlitre; EDTA: ethylenediaminetetraacetic acid; SES: socio-economic status; GEE: generalized estimating equation; Hb: haemoglobin; ACT: artemisinin-based combination therapy; PCR: polymerase chain reaction; CMP: Centre for Medical Parasitology; JMP: Joint Malaria Programme; NMCP: National Malaria Control Programme; NIMR: National Institute for Medical Research; MoHSW: Ministry of Health and Social Welfare; IQR: Inter-quartile range; SE: standard error; 95%CI: ninety five percent confidence interval; g/dL: grams per decilitre; GMPD: Geometric mean parasite density

## Competing interests

The authors declare that they have no competing interests.

## Authors' contributions

BPM participated in designing the study, data collection, data analysis, interpretation of results and manuscript preparation. MDS, DI, ASR and MS assisted in designing the study, data collection and manuscript preparation. HAM and SHS assisted in clinical assessment of clients. FF assisted in data management. MLK and MML participated in designing the study, supervision of field work, interpretation of results and manuscript development. All authors read and approved the manuscript.
